# Anti-Diabetes, Anti-Gout, and Anti-Leukemia Properties of Essential Oils from Natural Spices *Clausena indica*, *Zanthoxylum rhetsa*, and *Michelia tonkinensis*

**DOI:** 10.3390/molecules27030774

**Published:** 2022-01-25

**Authors:** Nguyen Van Quan, La Hoang Anh, Vu Quang Lam, Akiyoshi Takami, Rolf Teschke, Tran Dang Khanh, Tran Dang Xuan

**Affiliations:** 1 Transdisciplinary Science and Engineering Program, Graduate School of Advanced Science and Engineering, Hiroshima University, Hiroshima 739-8529, Japan; nvquan@hiroshima-u.ac.jp (N.V.Q.); hoanganh6920@gmail.com (L.H.A.); 2 Division of Hematology, Department of Internal Medicine, Aichi Medical University School of Medicine, Nagakute 480-1195, Japan; quanglamvu1991@gmail.com (V.Q.L.); takami.akiyoshi.490@mail.aichi-med-u.ac.jp (A.T.); 3 Division of Gastroenterology and Hepatology, Department of Internal Medicine II, Klinikum Hanau, Teaching Hospital of the Medical Faculty, Goethe University Frankfurt/Main, 63450 Hanau, Germany; rolf.teschke@gmx.de; 4 Agricultural Genetics Institute, Pham Van Dong Street, Hanoi 122000, Vietnam; khanhkonkuk@gmail.com

**Keywords:** anti-diabetes, anti-gout, anti-leukemia, antioxidant, essential oils, natural spices

## Abstract

Essential oils (EOs) of *Clausena indica* fruits, *Zanthoxylum rhetsa* fruits, and *Michelia tonkinensis* seeds were analyzed for their phytochemical profiles and biological activities, including anti-diabetes, anti-gout, and anti-leukemia properties. Sixty-six volatile compounds were identified by gas chromatography–mass spectrometry (GC–MS), in which, myristicin (68.3%), limonene (44.2%), and linalool (49.3%) were the most prominent components of EOs extracted from *C. indica*, *Z. rhetsa*, and *M. tonkinensis*, respectively. In addition, only EOs from *C. indica* inhibited the activities of all tested enzymes comprising α-amylase (IC_50_ = 7.73 mg/mL), α-glucosidase (IC_50_ = 0.84 mg/mL), and xanthine oxidase (IC_50_ = 0.88 mg/mL), which are related to type 2 diabetes and gout. Remarkably, all EOs from *C. indica*, *Z. rhetsa* (IC_50_ = 0.73 mg/mL), and *M. tonkinensis* (IC_50_ = 1.46 mg/mL) showed a stronger anti-α-glucosidase ability than acarbose (IC_50_ = 2.69 mg/mL), a known anti-diabetic agent. Moreover, the growth of leukemia cell Meg-01 was significantly suppressed by all EOs, of which, the IC_50_ values were recorded as 0.32, 0.64, and 0.31 mg/mL for EOs from *C. indica*, *Z. rhetsa*, and *M. tonkinensis*, respectively. As it stands, this is the first report about the inhibitory effects of EOs from *C. indica* and *Z. rhetsa* fruits, and *M. tonkinensis* seeds on the human leukemia cell line Meg-01 and key enzymes linked to diabetes and gout. In conclusion, the present study suggests that EOs from these natural spices may be promising candidates for pharmaceutical industries to develop nature-based drugs to treat diabetes mellitus or gout, as well as malignant hematological diseases such as leukemia.

## 1. Introduction

In recent years, studies on natural products from plants have received much attention from researchers all over the world, but difficulties remain for a plant-based product to be officially registered as a drug and widely applied in clinical practice if a comprehensive evaluation about benefits over risks is lacking [1]. The prerequisite for clinical randomized controlled trials (RCTs) is the prior preclinical discovery of biological activities among plant species that have been commonly used for a long time as foodstuffs and folk remedies. Several studies on food-based drugs have included the essential oils (EOs) from the natural spices from plant organs as potential candidates, with diverse biological and pharmacological activities [2,3]. 

In this context, of special interest are promising biological properties of EOs obtained from *Clausena indica, Zanthoxylum rhetsa,* and *Michelia tonkinensis* which have been popularly used as flavors and spices in many sub-tropical and tropical countries. More specifically, *C. indica* is widely distributed in South China, and South and Southeast Asia. Leaves and fruits of this plant have been widely used as a seasoning for many dishes in Vietnam [4]. Additionally, *C. indica*’s leaves and roots have shown therapeutic effects for many diseases. The essential oil from *C. indica* leaves was determined as having potential antimicrobial and antibacterial effects [5,6]. Bioactive components from *C. indica* roots were reported as having potential for antioxidants, anti-diabetes, and anti-aging via in vitro bioassays [4]. Similarly, *Z. rhetsa* is recognized as not only a spice, but also as a medicinal plant which has been popularly used in many tropical countries, such as Vietnam, Thailand, and Bangladesh [7]. Phytocompounds from parts of this plant were reported with extra health benefits, including antinociceptive and antidiarrheal [8], antioxidant, antimalarial, antimicrobial [9], and antibacterial [10] activities. In addition, EO from *Z. rhetsa* fruits has shown a preventative ability on several breast [11] and lung [12] cancer cell lines, as well as on leukemia [13]. On the other side, *M. tonkinensis* (syn. *Magnolia balansae*), is a medicinal plant, traditionally used to treat malaria, flu, and infections in Vietnam [14,15]. Hitherto, there has been little scientific information on the biological activity and biochemistry of this spice plant. Additionally, along with the medicinal purpose, the use of *C. indica*, *Z. rhetsa*, and *M. tonkinensis* as seasonings and condiments is a feature of the culinary culture of ethnic minorities in Northwestern Vietnam.

This study examines the antioxidant, anti-diabetic, anti-gout, and anti-leukemia properties of EOs from *Clausena indica*, *Zanthoxylum rhetsa*, and *Michelia tonkinensis* via in vitro studies. In addition, the composition of the individual EOs was identified using gas chromatography–mass spectrometry (GC–MS). 

## 2. Results

### 2.1. Phytochemical Composition of EOs

**Figure 1:**
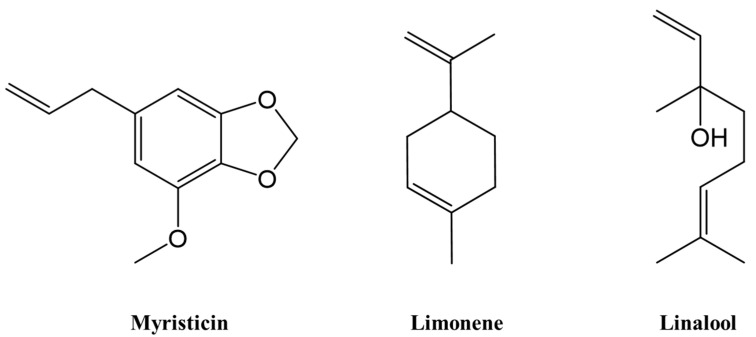
Structure of the major compounds of EOs from *C. indica*, *Z. rhetsa*, and *M. tonkinensis*.

By hydro-distillation, all essential oils from the natural spices were obtained with a pale-yellow color, of which, the EO extraction yields were 0.4, 1.7, and 5.5% for the CI, ZR, and MT samples, respectively (Table 1). Among 66 identified compounds, myristicin (68.3%) was the most dominant compound of CI oil, while limonene (44.2%) and linalool (49.3%) were the most prominent components of ZR and MT oils, respectively (Table 1 and Figure 1).

In terms of classification, benzodioxoles (myristicin) was the principal group of CI oil, followed by monoterpene hydrocarbons (14.4%), whereas the oxygenated monoterpene group (70.1%) was prominent in MT oil. Besides, the leading compounds of ZR oil were monoterpene hydrocarbons (65.5%) followed by oxygenated monoterpenes (26.0%).

### 2.2. Antioxidant Activity

**Figure 2:**
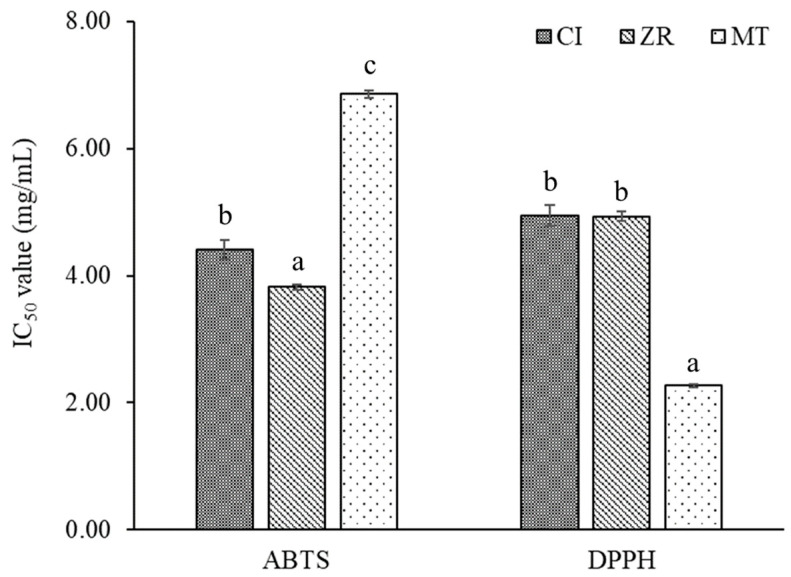
Antioxidant activities of essential oils extracted from *Clausena indica* (CI), *Zanthoxylum rhetsa* (ZR), and *Michelia tonkinensis* (MT). The letters represent the rank of antioxidant activity strength.

The antioxidant activity of CI, ZR, MT was determined via 2,2′-azino-bis(3-ethylbenzothiazoline-6-sulfonic acid) (ABTS) and 2,2-diphenyl-1-picrylhydrazyl (DPPH) assays (Figure 2). The results showed that EO from ZR (IC_50_ = 3.82 mg/mL) exerted the strongest antioxidant activity, followed by those of EO from CI (IC_50_ = 4.41 mg/mL) and MT (IC_50_ = 6.85 mg/mL), respectively, in terms of ABTS assay. In the DPPH assay, the most potent antiradical activity was exhibited by the EO from MT (IC_50_ = 2.27 mg/mL), followed by those of the CI (IC_50_ = 4.95 mg/mL) and ZR (IC_50_ = 4.93 mg/mL) oils. In general, EOs from CI, ZR, and MT performed with a negligible antioxidant activity in a comparison with that of the standard BHT (IC_50_ = 0.08 and 0.03 mg/mL for ABTS and DPPH assays, respectively).

### 2.3. Enzymatic Inhibitory Activity

The results indicating the inhibitory effect of EO samples on the target enzymes are presented in Table 2. 

Accordingly, only EO from CI exhibited the inhibition of α-amylase with an IC_50_ of 7.73 mg/mL, meanwhile, the activities of well-known anti-diabetic agents, acarbose and palmitic acid, were 0.01 and 1.57 mg/mL, respectively. On the other hand, all samples performed a considerable suppression of α-glucosidase, of which, EOs from ZR displayed the strongest activity with an IC_50_ of 0.73 mg/mL, followed by the EOs from CI (IC_50_ = 0.84 mg/mL) and MT (IC_50_ = 1.46 mg/mL). The EO from ZR had a similar α-glucosidase inhibition to that of palmitic acid (IC_50_ = 0.72 mg/mL). Remarkably, the α-glucosidase inhibition of all EO samples was significantly stronger than that of acarbose (IC_50_ = 2.69 mg/mL). 

In terms of the xanthine oxidase assay, the EO from CI expressed the most potent inhibitory activity (IC_50_ = 0.88 mg/mL), followed by the EOs from MT (IC_50_ = 1.73 mg/mL) and ZR (IC_50_ = 2.80 mg/mL) (Table 2). The anti-xanthine oxidase activities of EO samples were lower than that of allopurinol, an outstanding anti-gout drug (IC_50_ = 0.01 mg/mL). 

### 2.4. Cytotoxic Activity

**Figure 3:**
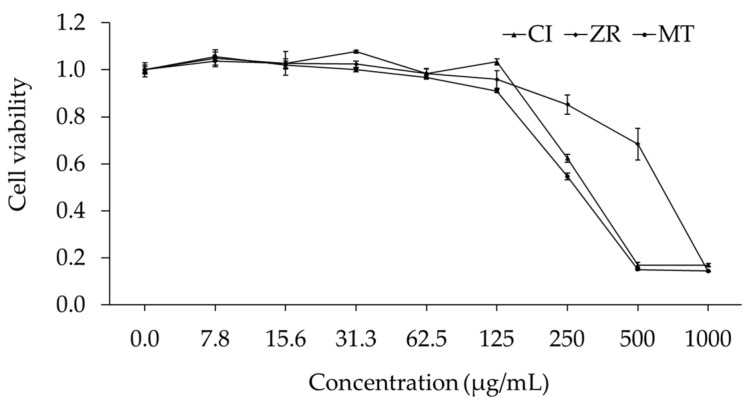
Dose–response curve for cytotoxic activity of essential oils from *Clausena indica* (CI), *Zanthoxylum rhetsa* (ZR), and *Michelia tonkinensis* (MT) against Meg-01 cell line.

In this research, in vitro MTT assay was used to evaluate the cytotoxicity of EO samples on the expansion of the Meg-01 cell line. The cytotoxic activity of EOs from CI, ZR, and MT against Meg-01 cell line is demonstrated in Table 2 and Figure 3. 

All samples manifested a significant inhibition of the growth of the Meg-01 cell line (Table 2 and Figure 3). Accordingly, stronger cytotoxicity was found in EOs of CI and MT which had IC_50_ values of 0.32 and 0.31 mg/mL, respectively, while lower activity was recorded in the EO of ZR, with an IC_50_ value of 0.64 mg/mL. Moreover, the dose–response curve shows a consistent result, of which, the EOs from CI and MT rapidly reduced the cell viability at concentrations ranging from 125 to 500 µg/mL; meanwhile, this point of ZR oil was from 250 to 1000 µg/mL (Figure 3). On the other hand, the standard anticancer drugs imatinib and doxorubicin exhibited stronger cytotoxicity on Meg-01, with IC_50_ values of 0.30 and 1.82 µg/mL, respectively.

## 3. Discussion

Numerous plant EOs have been used for therapeutic and cosmetic purposes throughout history, with special reference to monoterpenes that have been considered as their most dominant compounds. The biological benefits of some monoterpenes have been widely investigated [20]. Most monoterpenes may have antioxidant, anti-inflammatory effects [21]. Some valuable monoterpenes can be classified as β-myrcene, limonene, sabinene, terpinen-4-ol, linalool, and citral; β-myrcene can be found in numerous plant EOs. The safety of β-myrcene for human health has been widely confirmed. This volatile monoterpene showed potent antioxidant activity in the research of Ciftci et al. [22]. Additionally, β-myrcene can be used for osteoarthritis treatment [23] and as an alternative treatment after ischemic stroke [24]. Limonene is also an outstanding monoterpene, which can be easily perceived by its citrusy smell. This compound can be used to prevent many human disorders due to its anti-inflammatory, antioxidant, antiviral, anti-diabetic, and anticancer properties [25]. Another common monoterpene is sabinene, which has been acknowledged as having antimicrobial, antioxidant, angiostatic, and anti-angiogenic effects [26]. Terpinen-4-ol found in the EO from tea tree displayed antibacterial, antiviral, and antifungal capacities in previous studies [27]. Linalool is the most abundant monoterpene in lavender plants, which makes the flower rich in fragrance. The compound showed calming, anti-inflammatory, antimicrobial, neuroprotective, anti-depressant, anticancer, and anti-anxiety effects in previous studies [28]. Other monoterpenes consisting of citral with anti-inflammatory ability [29], and methyleugenol with antioxidant and antimicrobial activities [30], were also reported. Due to the valuable benefits of monoterpenes for human health, these compounds can be a promising source for developing natural remedies and supplements. In addition, their attractive color and scent is profitable for producing food-based drugs, which can be applied in aromatherapy to improve human health. Apart from monoterpenes, myristicin belonging to the group of benzodioxoles is a valuable compound found in the EOs of several plant species. Myristicin can be used to treat various diseases, for example, diarrhea, stomach aches and anxiety, Crohn’s disease, rheumatoid arthritis, and encephalomyelitis [31]. Besides, myristicin reveals hepatoprotective [32], antioxidant [33], anti-cholinergic [34], antibacterial [35], and especially anti-inflammatory properties via numerous biological pathways [36]. Based on myristicin’s biological benefits, together with its attractive color and fragrance, the plant Eos rich in myristicin content can be exploited for developing aromatherapy and functional supplements.

This study determines the EO composition of whole ripe fruits of *C. indica*, which is not only used as a flavoring but also applied as folk medicine in many South Asian countries. Moreover, this is the first report about chemical constituents of EO from CI fruits grown in northern Vietnam. In the research of John et al. [6], sabinene (53.1%), terpinen-4-ol (13.1%), γ-terpinene (5.0%) and β-phellandrene (4.5%) were the major compounds of the EO from fresh leaves of CI collected in India. Other studies on EO constituents extracted from plant parts of CI growing in Vietnam performed different results. In a study of Diep and collaborators [5], EO from the branches and leaves of CI contained myristicin (35.3%), terpinolene (16.7%), and δ-3-carene (11.3%) as the main constituents. Meanwhile, the other investigation on EO from CI leaves by Trung et al. showed 1-menthone (70.6%) and β-phellandrene (13.0%) were the most abundant compounds [14]. Thai et al. indicated that terpinolene (56.1%) and myristicin (17.9 %) were the most dominant compounds in EO from leaves and small fruits of CI [18]. In the case of *Z. rhetsa* fruits and seeds, they are well-known natural spices in many tropical countries. To date, the composition of EO from *Z. rhetsa* has been investigated, which varied from different plant parts and growing regions. EO from *Z. rhetsa* seeds collected from India contained sabinene as the major compounds [37], meanwhile, that of pericarp was terpinen-4-ol [38]. Theeramunkong and Utsintong found a difference between the EO components of fresh and dried ZR fruits grown in northern Thailand, in which limonene, β-phellandrene, and sabinene were the most dominant substances [12]. In the present study, EO from the whole ZR fruits consisted of limonene (44.2%) as the main component, followed by terpinen-4-ol (11.5%), and sabinene (4.8%), respectively. For *M. tonkinensis*, there have been limited studies on EO from this plant so far. Dai et al. reported that EO from MT leaves (in middle Vietnam) comprised α-pinene (40.3%), β-phellandrene (7.6%), and β-pinene (7.4%) as the primary compounds [15]. To the best of our knowledge, the present study is the first to investigate the EO composition of MT seeds. In particular, among the 21 identified compounds from the EO of MT seeds by GC–MS, linalool (49.3%), methyl eugenol (16.9%), α-citral (8.3%), and β-citral (5.6%) were the major phytocompounds of the sample collected in northern Vietnam. In general, the different results of the chemical profiles of CI, ZR, and MT essential oils, in previous and present studies, can be explained by the effects of various factors, such as climate, soil type, plant parts, growth stages, and especially extraction methods [20]. Since the chemical contents might determine the EO quantity and quality, appropriate collected samples and extraction methods can optimize the obtained EOs with multiple biological effects. 

Among the three natural spices, the antioxidant property of EO from ZR has been widely investigated. This is the first report of the antioxidant capacity of EOs from CI fruits and MT seeds. The results show various free radical scavenging abilities among samples. These observed differences can be attributed to the distinctive phytochemical components involved in each sample. Myristicin, a benzodioxole compound, was recognized as an antioxidant agent [33]. On the other side, the two monoterpenes limonene [39] and terpinen-4-ol [40] were noted with a considerable antioxidant property. Notably, Seol et al. demonstrated that the inhalation of linalool (a noncyclic monoterpenoid) could enhance antioxidative activity in patients with carpal tunnel syndrome [41]. Therefore, the combination of the principal compounds myristicin, limonene, and linalool with other constituents may contribute an important role to the antioxidant potentials of EOs from CI, ZR, and MT.

For human chronic disorders, the assessment of enzyme inhibition has been becoming a central target of current drug discovery and development [42]. Of the enzyme inhibitors, the natural-based inhibitors have attracted more interest in present clinical therapies because of their higher efficiency and safety compared with synthetic drugs. The present study examined the inhibitory capacities of Eos from CI, ZR, and MT on α-amylase, α-glucosidase, and xanthine oxidase, which are key enzymes related to type 2 diabetes and gout in humans. In our previous study, the CI fruits’ extract with a high content of myristicin showed a strong anti-α-amylase activity [43]. In addition, limonene and linalool performed significant antidiabetic activity via a streptozotocin-induced diabetic rat model [44]. Hence, myristicin, limonene, and linalool might possess a major role in the α-amylase inhibition of Eos from CI, ZR, and MT, respectively. In humans, α-amylase and α-glucosidase are the two major enzymes involved in carbohydrate digestion. These enzymes hydrolyze polysaccharides into smaller glucose molecules, which can be directly transferred to bloodstreams and result in postprandial hyperglycemia. In the current treatments for type 2 diabetic patients, most drugs and supplements are developed aiming to diminish the blood glucose level after meals through the suppressive action of α-amylase and α-glucosidase. Therefore, the use of EOs from the natural spices CI, ZR, and MT is promising for preventing the risk of type 2 diabetes, as well as having potential for the treatment of this chronic disease. 

In terms of hyperuricemia, xanthine oxidase participates in both uric acid production and oxidation. The over accumulated uric acid and free radicals in the body may lead to various problems, such as gout and cardiovascular diseases. Consequently, xanthine oxidase inhibitors have been regarded as a target enzyme and popularly applied for healing gout and oxidative stress [45]. This study, for the first time, revealed the xanthine oxidase inhibitory effect of EOs from CI and ZR fruits and MT seeds, which can be a paradigm for future dietetics and the further development of anti-gout drugs and antioxidants.

In addition, malignant hematological diseases are another serious disorder with an estimated 1.24 million cases that emerge annually in the world: their mortality rate accounts for 7% of global cancer deaths annually [46]. There are three main groups of malignant hematological disorders, including various forms of leukemia, lymphoma, and multiple myeloma. Meg-01 is a human megakaryoblastic leukemia cell line, which is a common model for examining the anti-chronic myeloid leukemia capacity. A previous study demonstrated that the ethanolic extract of ZR significantly inhibited the growth of a leukemia cell HL-60 [13], but the tested plant part was not mentioned. Recently, Poma et al. indicated that EO from *Euphorbia intisy* rich in terpenes showed significant cytotoxicity on the acute myeloid leukemia cell line HL-60 [47]. Notably, myristicin [48], limonene [49], and linalool [50] have proven cytotoxic properties on leukemic cells; therefore, these compounds may preferentially be responsible for the cytotoxic activities of EOs from CI, ZR, and MT on the Meg-01 cell in the present study. Furthermore, this is the first report about the cytotoxicity of EOs from CI and ZR fruits and MT seeds on a human leukemia cell line, Meg-01. Of major clinical interest is the current observation that issues of liver injury by CI, ZR, or MT are unknown, although the use of many herbal products and medicines carry the risk of liver injury according to worldwide analyses of published reports [51,52,53]. 

In humans, the pathogenesis of chronic diseases is attributed by some scientists to the endogenous imbalance between oxidant and antioxidant levels [54]. In particular, oxidative stress can lead to the exacerbation of inflammation, and vice versa; therefore, antioxidants may contribute to therapeutic solutions for numerous chronic illnesses [55]. The relationship between oxidative stress and the etiology and pathogeneses of hyperuricemia and blood glucose level has been proven by both laboratory and clinical studies [56]. Accordingly, uric acid, the final products of the purine hydrolysis by xanthine oxidase, can be an intracellular oxidant if at a high concentration. In the body, the increase in serum uric acid will result in oxidative stress, which directly or/and indirectly leads to a series of disorders such as metabolic syndrome and cancers. Realistically, chronic diseases such as diabetes mellitus mostly are multifactorial diseases, associated with complications and comorbidities [57]. Thus, the exploration of substances with simultaneous biological activities comprising antioxidant, anti-α-amylase, anti-α-glucosidase, anti-xanthine oxidase, and anti-Meg-01 cell, as shown in this present study, is essential in preventing and healing multiple serious diseases. In future studies, the mechanism of the anticancer activity as well as the bioavailability and bioaccessibility of the EOs from three natural spices should be elaborated. Additionally, although there have been no cases of poisoning reported when using these EOs, it is essential to investigate the toxicology and allergic potentials to determine the effective dose and dose-effect; especially, the tumor-selective property of these EOs should be further scrutinized. Although there are certain limitations to the phytochemical analysis, this research may contribute significant data to future metabolomic studies.

## 4. Materials and Methods

### 4.1. Instrumentations and Reagents

The composition of EOs was analyzed by the DB-5MS column (30 m × 0.25 mm × 0.25 μm, Agilent Technologies, J&W Scientific Products, Folsom, CA, USA) equipped with a GC–MS system (JMS-T100 GCV, JEOL Ltd., Tokyo, Japan), while biological activities were assayed by using a Multiskan^TM^ microplate reader (Thermo Fisher Scientific, Osaka, Japan) and SpectraMAX M5 spectrophotometer (Molecular Devices, Sunnyvale, CA, USA). Reagents, enzymes, and necessary chemicals were purchased from Fujifilm Wako Pure Chemical Corporation (Osaka, Japan), Fisher Scientific company (Hampton, NH, USA) and Sigma-Aldrich (St. Louis, MO, USA). The Meg-01 cell line was procured from the American Type Culture Collection (ATCC, Rockville, MD, USA).

### 4.2. Plant Materials 

The ripe fruits of *Clausena indica* (CI) and *Zanthoxylum rhetsa* (ZR) and seeds of *Michelia tonkinensis* (MT) were collected in Thai Nguyen and Bac Kan provinces, Vietnam, in September 2019. The identification of the plant species was conducted by the first author, based on the morphological characteristics used by the Vietnam Plant Data Center (http://www.botanyvn.com, accessed on 22 December 2021) and the United States (TROPICOS-http://www.tropicos.org, accessed on 22 December 2021) as major references. 

### 4.3. Essential Oil Extraction

All samples were sterilized by NaOCl (0.5%) solution within 1 h, and washed with distilled water several times. After draining, samples were dried in an oven at 35 ± 1 °C for 12 days. The dry samples were then separately subjected to the hydro-distillation system using a Clevenger apparatus for 6 h. The aqueous oil mixture was separated by diethyl ether with a ratio 1:2 *v*/*v*, three times. Subsequently, the organic phase was collected and evaporated under vacuum at 30 ± 1 °C to yield pure essential oils (EOs). EOs were preserved in individual screw-capped vials at 4 °C for further analyses. 

### 4.4. Identification of Phytochemical Composition by GC–MS

The chemical constituents of EOs were analyzed by the GC–MS system. The DB-5MS column, 30 m × 0.25 mm (0.25 μm film thickness) was the stationary phase, while helium was the mobile carrier at the split ratio 5:1. Initially, the EO sample (1 μL, 1 mg/mL in hexane) was injected into the GC system by an autosampler. The temperatures of the injection port and detector temperature were set at 300 °C and 320 °C, respectively. The oven temperature was programmed as follows: from 50 °C pushing up to 300 °C (10 °C/min), then isothermally maintaining for 20 min. The mass range was set at 29–800 amu. The EO components were identified based on their linear retention indices (LRI) and Kovats retention indices (KI) were determined by reference to a homologous series of *n*-alkanes (C7-C30), and by the comparison of their mass spectral fragmentation patterns with those reported in the literature. Additionally, the data analysis was performed by the JEOL’s GC–MS Mass Center System version 2.65a software that used NIST 20 as a mass spectral database. The available online databases of the National Center for Biotechnology Information, U.S. National Library of Medicine, Bethesda, MD, USA (PUBCHEM—https://pubchem.ncbi.nlm.nih.gov, accessed on 22 December 2021) and the National Institute of Standards and Technology, U.S. Department of Commerce (NIST- https://webbook.nist.gov, accessed on 22 December 2021) were used as well.

### 4.5. Evaluation of Biological Activities 

#### 4.5.1. Antioxidant Activity

The antioxidant capacities of the EOs were examined by DPPH radical scavenging and ABTS radical cation decolorization assays [4]. Briefly, a mixture of sample, 0.5 mM DPPH, and acetate buffer (0.1 M, pH 5.5) with a ratio of 2:1:1, *v/v* was incubated for 15 min at 25 °C in darkness. The resulting solution was scanned 517 nm by a microplate reader. In the ABTS assay, the working solution was prepared by diluting the 16 h-incubated mixture containing 7 mM ABTS and 2.45 mM potassium persulfate (1:1, *v/v*) in methanol. Afterwards, the EO sample and working solution (1:9, *v/v*) were homogenized, then incubated for 15 min at 25 °C in darkness. The absorbance was recorded at 734 nm. The radical scavenging percentage was determined by the following formula:

Radical scavenging activity (%) = [(A − B) − (C − D)]/(A − B) × 100(1)

where A is the absorbance of the reaction with MeOH as a negative control, B is the absorbance of the negative control without radical solution, C is the absorbance of the reaction with the EO sample or the positive reference, butylated hydroxytoluene (BHT), D is the absorbance of the tested sample without a radical solution.

#### 4.5.2. Enzymatic Assays

α-Amylase and α-glucosidase inhibitory assays were conducted following previous protocols with minor modifications [58] in a comparison with acarbose and palmitic acid as positive references. In particular, 20 μL of porcine pancreatic α-amylase (10 U/mL in phosphate saline buffer, pH 6.9) was mixed with 20 μL of EO sample. The mixture was then incubated at 37 °C for 10 min. The hydrolysis reaction was initiated by adding 30 μL of starch (0.5%). After 8 min-incubation at 37 °C, 20 μL of 1M HCl was added followed by 100 μL of 0.25 mM iodine solution. The absorbance was recorded at 565 nm. The inhibition of EOs on α-amylase was determined by the formula:

Inhibition percentage (%) = (E − Z)/(C − Z) × 100 (2)

where E is the absorbance of the reaction with EO samples, C is the absorbance of the reaction without α-amylase, and Z is the absorbance of the reaction without samples.

In the α-glucosidase assay, 20 μL of EO samples were blended with 0.1 M potassium phosphate buffer (pH 7.0) by a ratio of 1:2, *v/v* before incubation with 20 μL of enzyme (0.5 U/mL) at 25 °C for 5 min. Then, 20 μL of *p*-nitrophenyl-α-d-glucopyranoside (pNPG) substrate was added to activate the reaction. After 10 min-incubation at 25 °C, 100 μL of 0.1 M Na_2_CO_3_ was added to terminate the reaction. The absorbance was then read at 405 nm. The inhibition of EOs on α-glucosidase was gauged by the following formula:

Inhibition percentage (%) = (C − E)/C × 100 (3)

where C is the absorbance of the reaction without inhibitors, and E is the absorbance of the reaction with EO samples.

The inhibition of EOs on xanthine oxidase was examined by a method described by Bui et al. [59] with minor adjustments. In brief, a mixture of EO sample (20 μL), 70 mM phosphate buffer pH 7.5 (40 μL), and xanthine oxidase (0.05 U/mL, 40 μL) was merged and incubated at 25 °C for 8 min. After adding 60 μL of 300 μM xanthine to the mixture, an incubation at 25 °C was implemented for 15 min. Thereafter, 20 μL of 1M HCl was pipetted to the reaction and the absorbance of the final solution was recorded at 290 nm. The inhibitory effect of EOs on xanthine oxidase was calculated by the formula (3). Allopurinol was used as a positive control.

For each test, EO samples were prepared in a series of concentrations. IC_50_ value expressing 50% inhibitory capacity of a sample on a certain reaction was calculated by analyzing concentration versus percent inhibition via linear regression analysis. 

#### 4.5.3. Cytotoxic Assay on Meg-01 Cell Line

The inhibition of EOs on the growth rate of the Meg-01 cell line was determined by MTT assay described by Anh et al. [60]. Initially, the cells were cultured with 90 µL of a medium including fetal bovine serum (10%), L-glutamine (5 mM), penicillin (100 IU/mL), and of streptomycin (100 µg/mL) in a 96-well plate. The cultivation was incubated at 37 °C and 5% CO_2_ for 24 h, then 10 µL of EO samples with different dilution ranges were added to each well. After 48 h, 10 µL of MTT solution (5 mg/mL) was added to each well and the cells were incubated at 37 °C for 4 h. Afterwards, 100 µL of cell lysis buffer (10% SDS in 0.01 M HCl) was added to each well to dissolve the colored formazan crystals produced by MTT. A SpectraMAX M5 spectrophotometer (Molecular Devices, Sunnyvale, CA, USA) was used to measure the cell viability at 595 nm. Instead of EO samples, the culture medium (10 µL) was used as the negative control. The inhibition percentage of samples was calculated as formula (3) and IC_50_ value was achieved in the same way mentioned above. Imatinib and doxorubicin were used as standard anticancer drugs.

### 4.6. Statistical Analysis

The Minitab software version 16.0 (Minitab Inc., State College, PA, USA) was used for statistical analyses in this study. All analyses were thrice repeated. Data were displayed as mean ± standard deviation (SD). Significant differences among tests were determined by ANOVA (one-way) using Tukey’s test at *p* < 0.05.

## 5. Conclusions

The present study investigated the chemical composition and biological properties of EOs from natural spices *Clausena indica*, *Zanthoxylum rhetsa*, and *Michelia tonkinensis*. Myristicin, limonene, and linalool are identified as the major components of EOs from *C. indica*, *Z. rhetsa*, and *M. tonkinensis*, respectively, which may play an important role in their remarkable bioactivities. All EO samples exerted potential anti-α-amylase, anti-α-glucosidase, anti-xanthine oxidase, and cytotoxic activities. In the context of food-based drugs, which are becoming increasingly universally studied nowadays, results from this research may provide prospective candidates with information for the future development of novel drugs for type 2 diabetes, gout, and chronic myeloid leukemia. However, to better evaluate the mechanism, toxicology, and bioaccessibility of those natural spices, in vivo, pre-clinical, and clinical trials are necessary to be carried out before the commercial production.

## Figures and Tables

**Table 1:** Identified compounds in the essential oils of *Clausena indica*, *Zanthoxylum rhetsa*, and *Michelia tonkinensis*.

Peak No.	Identified Compound	Composition (%)			LRI	KI	Identification
		CI	ZR	MT			
1	β-Thujene	-	0.3	-	923	926	MS, ref
2	α-Pinene	0.1	2.2	-	933	936	MS, ref
3	5-Methylfurfural	0.1	-	-	955	959	MS, ref
4	Sabinene	-	4.8	0.1	973	976	MS, ref
5	6-Methyl-5-heptene-2-one	-	-	1.0	981	983	MS
6	β-Myrcene	6.5	1.2	-	985	987	MS, ref
7	α-Phellandrene	-	2.2	-	1009	1010	MS, ref
8	3-Carene	0.4	-	-	1008	1009	MS, ref
9	3,6-Dimethylene-1,7-octadiene	0.1	-	-	1013	1015	ref
10	α-Terpinene	-	1.4	-	1019	1021	MS, ref
11	*p*-Cymene	0.2	4.2	-	1023	1026	MS, ref
12	Limonene	5.5	44.2	-	1029	1031	MS, ref
13	β-Phellandrene	-	4.0	-	1034	1037	MS, ref
14	Eucalyptol	-	-	0.9	1036	1039	MS
15	1-Octanol	-	0.1	-	1068	1070	MS, ref
16	*p*-Cresol	0.1	-	-	1066	1069	MS, ref
17	*cis*-Linalool oxide	-	-	0.8	1072	1074	MS
18	*cis*-Linaloloxide	-	-	0.8	1088	1089	MS
19	α-Terpinolene	1.6	1.1	-	1084	1086	MS, ref
20	*p*-Cymenene	1.2	0.1	-	1088	1090	MS, ref
21	Linalool	-	1.3	49.3	1099	1101	MS
22	6-Camphenone	0.3	-	-	1095	1095	ref
23	1,5,7-Octatrien-3-ol, 3,7-dimethyl-	-	-	0.3	1103	1104	ref
24	1,3,8-*p*-Menthatriene	0.1	-	-	1111	1112	MS, ref
25	*cis*-*p*-Mentha-2,8-dienol	-	0.2	-	1124	1126	ref
26	2-Cyclohexen-1-ol, 1-methyl-4-(1-methylethyl)-, *trans*-	-	0.5	-	1127	1129	ref
27	*trans*-*p*-Menth-2-en-1-ol	0.2	0.3	-	1138	1141	MS, ref
28	Pinocarveol	-	0.3	-	1145	1148	ref
29	1,5,7-Octatrien-3-ol, 2,6-dimethyl-	0.4	-	-	1151	1154	MS, ref
30	*E*-β-Terpineol	-	0.2	-	1160	1163	ref
31	Octanoic acid	-	0.3	-	1164	1166	ref
32	2-Isopropenyl-5-methylhex-4-enal	0.8	-	-	1178	1180	MS, ref
33	Terpinen-4-ol	-	11.5	0.9	1184	1186	MS, ref
34	*p*-Cymen-8-ol	1.6	2.8	-	1188	1189	MS, ref
35	α-Terpineol	0.5	2.3	0.4	1197	1198	MS, ref
36	*cis*-Carveol	0.1	1.8	-	1221	1222	MS, ref
37	Citronellol	-	-	1.7	1225	1226	MS
38	*cis*-*p*-mentha-1(7),8-dien-2-ol	0.1	-	-	1228	1229	MS, ref
39	Nerol	-	0.3	-	1234	1236	ref
40	β-Citral	-	-	5.6	1238	1240	MS
41	Phenol, 2-ethyl-4,5-dimethyl-	0.1	-	-	1240	1242	MS, ref
42	Carvone	-	3.3	-	1247	1249	ref
43	*trans*-Geraniol	-	-	3.8	1249	1251	MS
44	Nonanoic acid	-	0.2	-	1260	1262	MS
45	α-Citral	-	-	8.3	1267	1269	MS
46	Phellandral	-	0.4	-	1282	1283	MS
47	*p*-Cymen-7-ol	-	0.3	-	1292	1293	MS, ref
48	Safrole	-	-	4.6	1292	1293	MS
49	*p*-Cymen-2-ol	-	0.3	-	1297	1297	MS, ref
50	Geranic acid methyl ester	-	-	0.1	1319	1320	MS
51	*p*-Mentha-1,4-dien-7-ol	-	0.2	-	1329	1331	MS, ref
52	Eugenol	0.1	-	-	1349	1350	MS, ref
53	2,6-Octadien-1-ol, 3,7-dimethyl-, acetate	-	1.4	-	1375	1377	MS, ref
54	Methyleugenol	0.4	-	16.9	1399	1399	MS, ref
55	Caryophyllene	0.6	-	0.2	1428	1429	MS, ref
56	*trans*-α-Bergamotene	0.1	-	-	1433	1434	MS, ref
57	*cis*-β-Famesene	0.1	-	-	1448	1449	MS, ref
58	β-Eudesmene	-	-	0.4	1497	1497	MS
59	β-Bisabolene	2.6	-	0.1	1510	1510	MS, ref
60	δ-Cadinene	-	-	0.2	1522	1523	MS
61	Myristicin	68.3	-	-	1524	1525	MS, ref
62	Elemicin	1.9	-	-	1540	1542	MS, ref
63	Spathulenol	0.2	0.1	-	1581	1582	MS, ref
64	Isoelemicin	0.1	-	-	1639	1640	MS, ref
65	α-Cadinol	-	-	0.2	1661	1662	MS
66	α-Springene	2.9	-	-	1964	1965	MS, ref
Monoterpene hydrocarbons		14.4	65.5	0.1			
Oxygenated monoterpenes		2.5	26.0	70.1			
Sesquiterpene hydrocarbons		3.4	0	0.9			
Oxygenated sesquiterpenes		0.2	0.1	0			
Others		76.6	1.9	25.5			
Total identified		96.9	93.5	96.6			
Yield % (*w*/*w*)		0.36	1.69	5.49			

CI, *Clausena indica*; ZR, *Zanthoxylum rhetsa*; MT, *Michelia tonkinensis*; LRI and KI, linear retention index and Kovats index calculated from DB5-MS column against *n*-alkanes (Figure S1); MS, identified based on matching the mass spectra with those from NIST 20 library; ref, identification based on comparing calculated retention indices with those reported from the literature [5,6,14,15,16,17,18,19].

**Table 2:** Inhibitory activities of essential oils extracted from *Clausena indica*, *Zanthoxylum rhetsa*, and *Michelia tonkinensis* on α-amylase, α-glucosidase, xanthine oxidase and Meg-01 cell line.

Sample	IC_50_ (mg/mL)			
	α-Amylase Assay	α-Glucosidase Assay	Xanthine Oxidase Assay	Meg-01 Assay
CI	7.73 ± 0.10	0.84 ± 0.03 ^b^	0.88 ± 0.05 ^a^	0.32 ± 0.01 ^a^
ZR	-	0.73 ± 0.01 ^a^	2.80 ± 0.14 ^c^	0.64 ± 0.04 ^b^
MT	-	1.46 ± 0.01 ^c^	1.73 ± 0.16 ^b^	0.31 ± 0.01 ^a^
Acarbose	0.01 ± 0.00	2.69 ± 0.07	nd	nd
Palmitic acid	1.57 ± 0.04	0.72 ± 0.01	nd	nd
Allopurinol	nd	nd	0.01 ± 0.00	nd

Data are expressed as mean ± standard deviation (n = 3); dissimilar superscript letters in the same column show significant differences among tested samples at *p* < 0.05; -, no effect; nd, not determined; CI, *Clausena indica*; ZR, *Zanthoxylum rhetsa*; MT, *Michelia tonkinensis*.

## Data Availability

All data are presented in the article.

## Supplementary Materials

The following are available online, Figure S1: Total ion chromatogram of n-alkanes.
