# Supplementary material for: Anti-Diabetes, Anti-Gout, and Anti-Leukemia Properties of Essential Oils from Natural Spices Clausena indica, Zanthoxylum rhetsa, and Michelia tonkinensis
Source: Molecules. 2022 Jan 25;27(3):774. doi: 10.3390/molecules27030774 (PMC8840550; doi:10.3390/molecules27030774)
Supplement: Supplementary file 1 [file molecules-27-00774-s001.zip › molecules-1547029-supplementary.pdf]

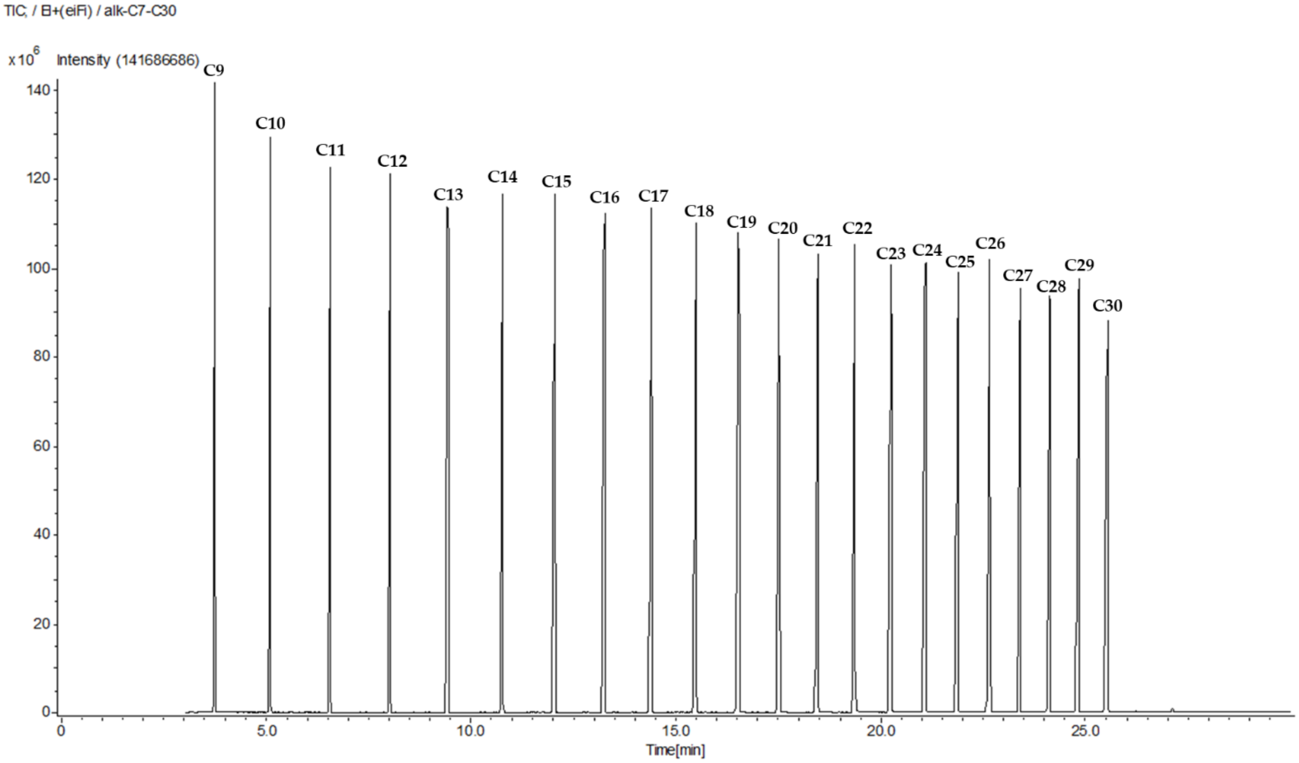

Figure S1. Total ion chromatogram of *n*-alkanes (C7-C30)

(*n-C7 and n-C8 are not presented due to the setup of retention program*)
